# Iatrogenic Superior Vena Cava Syndrome after Cardiopulmonary Bypass Diagnosed by Intraoperative Echocardiography

**DOI:** 10.1155/2020/8813065

**Published:** 2020-08-18

**Authors:** Matthew B. Ellison, Alex Statler, Roy E. Henrickson, Julia Graff, Daniel Sloyer, Mir Ali Abbas Khan, Heather K. Hayanga, Pavithra R. Ellison

**Affiliations:** ^1^Division of Cardiovascular and Thoracic Anesthesiology, Department of Anesthesiology, West Virginia University School of Medicine, P.O. Box 8255, 1 Medical Center Drive, Morgantown, WV 26506, USA; ^2^Department of Anesthesiology, West Virginia University School of Medicine, P.O. Box 8255, 1 Medical Center Drive, Morgantown, WV 26506, USA; ^3^West Virginia University School of Medicine, 1 Medical Center Drive, Morgantown, WV 26506, USA

## Abstract

A 73-year-old female patient presented for mitral valve replacement and coronary artery bypass grafting secondary to multivessel coronary disease and severe mitral valve regurgitation with moderate stenosis. After bypass, the patient developed refractory hypotension with decreased biventricular volume and elevated central venous pressure (CVP). Transesophageal echocardiography (TEE) was utilized to make the diagnosis of acute intraoperative superior vena cava (SVC) syndrome. The SVC cannulation site was revised, resulting in resolution of the hypotension and a decrease in the CVP. Intraoperative TEE was vital in recognizing, managing, and ultimately repairing the acute intraoperative SVC stenosis.

## 1. Introduction

Superior vena cava (SVC) syndrome is loosely recognized as a group of signs and symptoms resulting from obstruction of blood flow through the SVC. Most of the venous blood from both arms and the head, neck, and upper thorax drains via the SVC. Obstruction of this vessel may lead to edema of the neck, head, larynx, and face which in turn may cause dyspnea at rest, cough, and headache [1]. Most cases of SVC syndrome are caused by thoracic malignancies, but there are an increasing number of cases due to benign causes, primarily via thrombosis secondary to semipermanent catheter or pacemaker placement. SVC syndrome following pacemaker placement often occurs secondary to the formation of vegetations or via thrombosis after endothelial disruption [2, 3]. It has been found that up to 40% of SVC syndrome cases are due to benign causes that are most often iatrogenic [4]. Additionally, most of the reported instances of SVC syndrome develop chronically or semiacutely secondary to malignancy, fibrosis, or thrombosis. Only in rare cases have rapidly developing acute SVC syndromes been described. One such report describes an intraoperative occurrence secondary to a left internal thoracic artery retractor mechanically compressing the SVC [5]. In this case, we report an unusual instance of acute SVC syndrome secondary to stenosis caused by closure of the superior venous cannula used during cardiopulmonary bypass.

## 2. Case Report

A 73-year-old woman presented for mitral valve replacement and coronary artery bypass grafting. The patient had symptomatic severe mitral valve regurgitation and moderate mitral valve stenosis along with significant multivessel coronary disease. Her other past medical history included hypothyroidism, a long smoking history, and preserved left ventricular function on echocardiogram.

**Figure 1:**
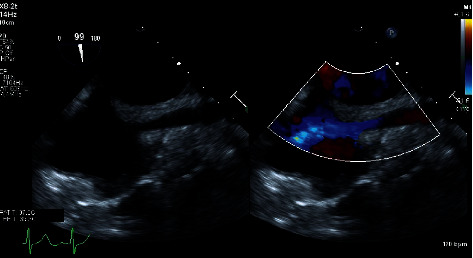
The image in the left panel shows a two-dimensional presurgical midesophageal bicaval view obtained via transesophageal echocardiography (TEE) which demonstrates mild narrowing of the SVC at the SVC-right atrial junction. The image in the right panel is the same with the addition of color flow Doppler demonstrating no flow acceleration from the superior vena cava into the right atrium.

After transportation to the operating room and placement of a left radial arterial line, anesthetic induction utilizing intravenous midazolam 2 mg, fentanyl 200 mcg, lidocaine 2% 80 mg, propofol 100 mg, and rocuronium 100 mg was performed. Following this, a right internal jugular 9-French multiaccess catheter (MAC Two-Lumen Central Venous Access Cath-Gard; Teleflex, Morrisville, NC) was placed under direct ultrasound guidance without complication. Next, an adult transesophageal echocardiography (TEE) probe was placed, and a full intraoperative exam was performed. Mild narrowing of the SVC at the SVC-right atrial (RA) junction was recognized on the midesophageal bicaval TEE view with no flow acceleration by color flow Doppler (Figure 1).

The patient underwent sternotomy, exposure, and takedown of the left internal mammary artery (LIMA) without issue. An aortic cannula was placed over the wire utilizing TEE guidance, and bicaval venous cannulas were utilized. A 24-French single-stage right angle cannula was placed in the SVC (Edwards Thin-Flex 90 degree 24 French Venous Cannulae; Edwards Lifesciences, Irvine, CA). After initiation of cardiopulmonary bypass, good flow was obtained from both venous cannulas. The patient underwent three coronary artery bypass grafts including the LIMA to the left anterior descending artery, a right saphenous vein graft to the obtuse marginal 1, and a right saphenous vein graft to the distal right coronary artery. Next, a chordal-sparing mitral valve replacement with a 25 mm bioprosthetic valve (EPIC Mitral Stented Tissue Valve; Abbott Structural Heart, Santa Clara, CA) was successfully performed.

**Figure 2:**
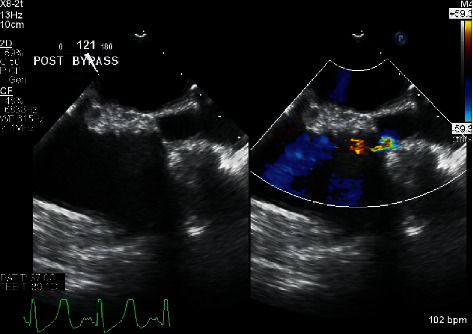
The image in the left panel shows the initial postbypass two-dimensional presurgical midesophageal bicaval TEE view demonstrating superior vena cava stenosis. The image in the right panel is the same with the addition of color flow Doppler demonstrating flow acceleration across the stenosis.

**Figure 3:**
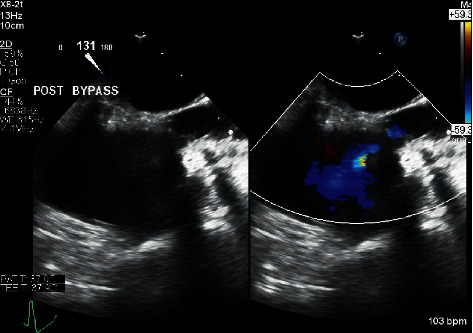
The image in the left panel shows the two-dimensional midesophageal bicaval TEE view after surgical revision. The image in the right panel is the same with the addition of color flow Doppler demonstrating improved flow from the SVC into the right atrium.

After successfully weaning from bypass, the intraoperative TEE showed no paravalvular leak, no regurgitant jet, and no stenosis of the replaced mitral valve with a mean transvalvular gradient of 3 mm Hg. Immediately following separation, the patient had normal biventricular function and volume with minimal vasopressor support. The venous and aortic cannulas were removed, and protamine was administered. Shortly thereafter, the patient became progressively more hypotensive, requiring initiation and escalation of vasopressors. An epinephrine infusion was started at 0.03 mcg/kg/min and titrated to 0.06 mcg/kg/min, and a norepinephrine infusion was initiated at 0.06 mcg/kg/min. In addition, multiple doses of intravenous phenylephrine 100 mcg and vasopressin 1 unit were administered. At this point, the intraoperative TEE demonstrated decreased biventricular volumes with normal systolic and valvular function. Intravenous fluid and 5% albumin were simultaneously administered along with the vasopressors. The central venous pressure had increased from approximately 15 mm Hg after bypass to 28 mm Hg. The hypotension was refractory to volume loading and vasopressor medications. A more thorough TEE exam showed significant flow acceleration at the SVC-RA junction distal to the superior venous cannulation site (Figure 2) as well as continually diminished ventricular volumes despite volume loading. Based on the TEE exam and the clinical picture, the surgeon and anesthesiologist determined that the narrowing at the SVC-RA junction was likely caused by the SVC cannulation site closure and that surgical repair was warranted. Additional heparin was given, vascular clamps were placed proximally and distally on the SVC, and the purse string was removed and the vessel closed in a transverse linear fashion. After repair, the CVP decreased from 28 mm Hg to 18 mm Hg and the vasopressor requirements were substantially decreased. The TEE demonstrated significantly improved biventricular volumes and less flow acceleration and narrowing at the SVC-RA junction (Figure 3). The remainder of the case proceeded uneventfully, and the patient was transported to the intensive care unit. She was extubated several hours later while on no vasopressor support.

## 3. Discussion

The superior vena cava carries approximately one-third of the venous return. When it is obstructed, a collection of symptoms that include swelling of the face and neck along with dilation of subcutaneous vessels in the upper extremity and chest, cyanosis, and plethora occur in what is termed SVC syndrome. Over time, blood will flow to the collateral venous circulation, primarily the azygos system, which can take weeks to expand to contain the extra blood supply [6, 7]. Reverse Trendelenburg positioning has been described as safe and effective in aiding venous drainage through this collateral system. However, it is more effective in the chronic cases when the system has had time to expand [8]. Reverse Trendelenburg positioning can be used in acute cases to maintain cardiac output while attempting to locate and alleviate the offending obstruction. In the case of acute or rapid onset such as those from thrombosis, the lack of collateral circulation can lead to life-threatening situations, including cerebral and laryngeal edema [9]. Additionally, an elevated cervical venous pressure as high as 20–40 mm Hg (compared with the normal of 2–4 mmHg) is seen [6]. SVC syndrome is frequently diagnosed through the presence of clinical symptoms as well as the use of imaging modalities. Computerized tomography of the chest after administration of contrast, positron-emission tomography, and transesophageal echocardiography have all been found to be useful in diagnosing SVC syndrome [6, 10, 11].

A review to determine the etiology of SVC syndrome found that the majority of benign cases are iatrogenic. These most often occur secondary to thrombosis associated with an intravascular device, catheter, or pacemaker wire [4, 5]. Perioperative cases have been documented resulting from laceration of the SVC vessel during surgery [10]. However, perioperative SVC syndrome has rarely been discussed due to stenosis secondary to closure of a venous cardiopulmonary bypass cannula. In this case, when the cannulation site was closed with the pledgetted purse-string suture, it resulted in tension on the main lumen yielding an iatrogenic SVC stenosis. This occurred distal to the SVC location where the CVP was being transduced, resulting in a CVP that did not accurately reflect right ventricular (RV) preload. The purse-string suture created a pressure gradient between the SVC and RA. The RA was not filling from the head, neck, and upper thorax, resulting in low RV volume. This scenario would explain the persistent low volume in RA, RV, and left ventricle despite a significantly elevated CVP. With volume loading, the patient was still unable to get suitable flow past the stenotic site and was just elevating the pressure proximal to the site which was evidenced by a CVP of 34 mm Hg. Although the postrevision flow acceleration at the SVC-RA junction was still greater than baseline on TEE, the patient's acute condition was significantly improved as evidenced by increased biventricular volumes and decreased vasopressor requirements.

In summary, we have described what we believe is a previously unreported cause of iatrogenic SVC syndrome secondary to closure of the superior venous cannulation site in a bicaval cannulation strategy. Patients who experience decreased biventricular volume, elevated CVP, and refractory hypotension following cardiopulmonary bypass should be evaluated for SVC stenosis utilizing the TEE midesophageal bicaval view. In this case, we were able to compare the patient's prebypass TEE imaging with that obtained immediately after bypass to make a timely potentially life-saving diagnosis. This report highlights the importance of not only performing a thorough TEE exam at each stage of the surgical procedure but also the necessity of archiving those images.

## Data Availability

No data were used to support this study.

## Abbreviations

SVC: Superior vena cava
TEE: Transesophageal echocardiography
CVP: Central venous pressure
LIMA: Left internal mammary artery
RA: Right atrium
RV: Right ventricle.
