# Corrigendum to “Iatrogenic Superior Vena Cava Syndrome after Cardiopulmonary Bypass Diagnosed by Intraoperative Echocardiography”

**DOI:** 10.1155/2020/8179176

**Published:** 2020-10-13

**Authors:** Matthew B. Ellison, Alec Statler, Roy E. Henrickson, Julia Graff, Daniel Sloyer, Mir Ali Abbas Khan, Heather K. Hayanga, Pavithra R. Ellison

**Affiliations:** ^1^Division of Cardiovascular and Thoracic Anesthesiology, Department of Anesthesiology, West Virginia University School of Medicine, P.O. Box 8255, 1 Medical Center Drive, Morgantown, WV 26506, USA; ^2^Department of Anesthesiology, West Virginia University School of Medicine, P.O. Box 8255, 1 Medical Center Drive, Morgantown, WV 26506, USA; ^3^West Virginia University School of Medicine, 1 Medical Center Drive, Morgantown, WV 26506, USA

In the article titled “Iatrogenic Superior Vena Cava Syndrome after Cardiopulmonary Bypass Diagnosed by Intraoperative Echocardiography” [1], there was a spelling error in author Alec Statler's name in the authors' list, where Alex Statler should have read Alec Statler. This is corrected as shown above.
